# Environmental drivers of taste characteristics in wild edible plants: Effects of topographic gradients and human harvesting evaluated using dwarf bamboo culms

**DOI:** 10.1371/journal.pone.0360962

**Published:** 2026-10-28

**Authors:** Noboru Katayama, Noriko Ryuda, Masasuke Ryuda, Kentaro Takagi

**Affiliations:** 1 General Education, Faculty of Commerce, Otaru University of Commerce, Otaru, Hokkaido, Japan; 2 Analytical Research Center for Experimental Sciences, Saga University, Saga, Japan; 3 Teshio Experimental Forest, Field Science Center for Northern Biosphere, Hokkaido University, Horonobe, Hokkaido, Japan; University of Pernambuco: Universidade de Pernambuco, BRAZIL

## Abstract

Wild edible plants grow in natural environments with little direct human management; consequently, their productivity and quality frequently exhibit pronounced spatiotemporal variability, making stable supply difficult. This variability represents a major constraint on the exploitation of wild edible plants as local food resources, and overcoming it requires an ecological understanding of the factors affecting variation in both productivity and quality. Here, we examined the effects of human harvesting and environmental factors on the taste characteristics of young culms of *Sasa kurilensis*. No statistically significant differences were detected between harvested and control plots in any major taste index, including umami, bitter aftertaste, astringency, umami richness, and sweetness (measured as Brix values). In contrast, taste characteristics showed substantial variation among study plots, suggesting a strong association with local environmental conditions. Elevation and slope angle were significantly correlated with bitter aftertaste, umami, and sweetness; plots at higher elevations and on steeper slopes tended to produce plants with a less bitter aftertaste, and higher sweetness and umami. Principal component analysis integrating multiple taste indices revealed that the major axis of taste variation was significantly associated with elevation, and that harvesting treatment had no detectable effect. These results suggest that the taste characteristics of young culms in the study area are governed primarily by topographic gradients rather than by human harvesting. Although wild edible plants are important food resources in boreal and temperate forests worldwide, many are poorly managed. By quantitatively establishing relationships between taste characteristics and environmental factors in young culms, this study provides a scientific basis for determining the optimal timing, location, and selection criteria for wild edible plant harvesting, thereby contributing to the development of sustainable use strategies.

## Introduction

Forest ecosystems have long provided humans with a wide range of services that are deeply intertwined with human life, including food and material resources, climate regulation, and cultural and recreational values [1,2]. Among these services, wild edible plants obtained directly from forest ecosystems represent a tangible example of the provisioning function of forests through their role as food resources [3–5]. The harvesting of wild edible plants is a food acquisition strategy that predates agriculture and cultivation, and has long been closely associated with human diets, cultures, and local livelihoods in tropical, temperate, and boreal forest regions worldwide [6–8]. In recent years, the harvesting of wild edible plants has also been recognized as a recreational activity that allows people to experience interactions with nature, serving both as a form of experiential learning that promotes ecological knowledge and awareness of sustainable use [9] and as a resource for experiential tourism [10]. Consequently, its value as a cultural ecosystem service is increasing.

Unlike agricultural crops, wild edible plants are wild resources that are rarely subject to deliberate cultivation or management. As a result, their productivity is strongly influenced by environmental conditions and interannual variability at their growth sites, leading to substantial spatiotemporal variation [11,12]. This instability represents a major constraint for the sustainable use and management of wild edible plants as ecosystem services and highlights the challenge of ensuring stable and sustainable access to these resources [9,13,14]. Quantitatively understanding the factors that affect the productivity of wild edible plant resources is therefore an important research priority, with implications for the evaluation and monitoring of ecosystem services and for sustainable resource management. Environmental conditions may also influence the quality of these resources, although this aspect has received far less attention.

Food quality is one of the key attributes determining the value of wild edible plants as food resources because it directly influences their acceptability and consumer preference. Mountain forest ecosystems, which provide habitats for wild edible plants, are characterized by continuous environmental gradients such as elevation, slope conditions, and soil moisture, which generate spatial heterogeneity in microclimate and resource availability within forests [15]. These gradients may influence not only quantitative traits, such as plant growth and morphology, but also qualitative traits through their effects on plant phytochemical composition and resource allocation [16], which may in turn affect taste and palatability.

In addition, the quality of wild edible plants may be influenced not only by their growing environment but also by anthropogenic management and harvesting. It is well established that, in many plant species, herbivory by insects and other herbivores induces defensive responses that alter the chemical composition of plant tissues, including secondary metabolites [17,18]. Because some of these induced responses can also be triggered by mechanical wounding [18,19], harvesting, which involves the removal of plant tissues, may likewise affect the chemical traits of plants and, consequently, the taste characteristics of edible plant parts. A previous study on the edible young culms of dwarf bamboo, a wild edible plant, used sensory evaluation to examine the effects of harvesting on the taste of newly produced edible shoots and found no significant effect of harvesting [20]. However, few studies have quantitatively examined how harvesting influences the taste characteristics of wild edible plants. Furthermore, to the best of our knowledge, no study has evaluated the effects of both harvesting and environmental conditions on taste characteristics within the same analytical framework or compared their relative importance. To address this knowledge gap, we conducted a field study using an edible dwarf bamboo as a study system.

Dwarf bamboos (*Sasa* spp.), which are widely distributed in boreal and cool-temperate forests, are dominant components of the forest understory in East Asian forest ecosystems [21,22]. They also play important roles in understory vegetation, nutrient cycling, and forest regeneration [23–25]. Their high productivity [26] and strong regenerative ability following harvesting [20,27] make them valuable wild plant resources with potential for sustainable use [28,29]. In addition, young culms that emerge in spring are widely consumed as seasonal foods in East Asia [27,30]. Previous studies on edible young *Sasa* culms have primarily focused on productivity and morphological responses to harvesting and environmental conditions [20,27,31], providing important insights into the ecological factors influencing resource production and regeneration. These ecological characteristics, together with their importance as a wild edible resource, make *Sasa* a suitable model species for investigating the environmental and anthropogenic drivers of quality variation in wild edible plants. However, taste characteristics have received far less attention, and the use of edible young *Sasa* culms has largely relied on empirical knowledge rather than systematic evaluation. Consequently, systematic resource management or commercialization comparable to that of agricultural crops has rarely been developed.

The objective of this study was to examine the effects of harvesting and environmental factors, particularly elevation and slope angle, on the taste characteristics of edible young culms of *Sasa kurilensis* (Rupr.) Makino & Shibata, a dwarf bamboo species widely distributed in the forests of northern Japan, and to evaluate their respective contributions in explaining variation in individual taste attributes. We predicted that the relative contributions of harvesting and environmental conditions would differ among taste attributes. To quantitatively evaluate taste characteristics, we employed a taste sensing system that provides objective and reproducible measurements of multiple taste attributes based on the responses of lipid membrane sensors to chemical compounds associated with different taste modalities [32–34]. This approach enables statistical evaluation of the effects of environmental conditions and harvesting treatments on individual taste attributes. By testing these predictions, this study introduces taste as a quantitative dimension into *Sasa* research, which has traditionally focused on quantitative traits such as culm number and culm diameter, and provides a new perspective for comprehensively assessing the resource value of edible young culms from both productivity and quality perspectives. These findings are expected to provide a scientific basis for the sustainable use of wild edible plants and their management as regional resources.

## Materials and methods

### Study site and sample collection

This study was conducted in the Teshio Experimental Forest of Hokkaido University (44°54′–45°06′N, 141°56′–142°10′E; area: 22,517 ha). The forest understory at the study site is dominated by *Sasa senanensis* (Franch. & Sav.) Rehder and *S. kurilensis* [27]. The study plots used in this study are existing plots that have been monitored continuously since 2013 to evaluate the productivity, morphological traits, and effects of harvesting on *S. kurilensis* [31] (S1 Fig and S2A Fig). Since 2019, all emerging bamboo shoots within each plot have been harvested annually in half of the plots. Since the plots were originally established in 2013 (n = 50), six plots had been lost by 2023 due to treefall or forestry operations. Young culm abundance recorded in 2013–2015, before the harvesting experiment was initiated in 2019, did not differ significantly between plots subsequently assigned to the harvested and control treatments (S1 Table).

Edible culms of young *S. kurilensis* were collected at each site between 21 and 30 May 2024 (S2B Fig). Sampling was conducted twice at intervals of 4–6 days because the timing of culm emergence varied both among and within sites, resulting in differences in the optimal harvesting period. Sampling at each site was conducted when young edible culms reached a stage suitable for harvesting.

Individual study plots (2 m × 2 m) were demarcated with pink tape. Edible young culms were collected by breaking them off at the base, following standard harvesting practices [27]. In harvesting plots, 18.14 ± 9.57 young culms (mean ± SD; range: 4–33 culms; n = 22 plots) were harvested within each plot (S1 Dataset). In control plots, no young culms were harvested within the plots, thereby maintaining the plots as unharvested controls for evaluating harvesting effects in future studies; instead, 7–14 young culms were selected and collected from the surrounding area for sample preparation.

This field study was conducted with permission from the Teshio Experimental Forest, Field Science Center for Northern Biosphere, Hokkaido University, which manages the study site. The study site is not privately owned and is managed as a university research forest. No protected species were sampled, and the study complied with all relevant institutional and national regulations.

### Pretreatment and storage

Before processing, collected culms that were clearly unsuitable for consumption due to decay or herbivore damage were excluded. The remaining samples were trimmed to a maximum length of approximately 30 cm with the leaf sheaths attached. Longitudinal incisions were made in the leaf sheaths, and the samples were parboiled immediately; these incisions facilitated removal of the leaf sheaths after boiling.

After boiling, the samples were cooled under running water and the leaf sheaths removed. The edible portions were then cut to fit into heat-resistant wide-mouth glass jars (height: 12.5 cm; diameter: 7 cm; with tin lids) and sealed (S2C Fig). The jars were placed in a pot, filled with water up to the jar openings, and boiled for 30 min. Immediately after boiling, the lids were tightly closed, and hermetically sealed with vinyl tape. The jars were then transported to the laboratory at Otaru University of Commerce and stored under refrigerated conditions (ca. 5 °C) until taste analysis.

### Measurement of taste characteristics

The stored jar samples were opened one week prior to analysis, transferred into sealed plastic bags, and transported under frozen conditions to Saga University, where they were stored at –20°C until analysis.

For taste analysis, one sample was prepared from the stored material derived from each study plot, and each plot was treated as one analytical unit. For sample preparation, 170 mL of Milli-Q water was added to 85 g of frozen sample (total 255 g), and the mixture was homogenized twice for 30 s using a food processor (TK441, TESCOM Co., Ltd., Tokyo, Japan). The sample-preparation procedure was based on a protocol recommended by the taste-sensor manufacturer following preliminary testing; aqueous homogenization of bamboo shoots has also been used in previous taste-sensing analyses [35]. The resulting suspension was then divided into three 50 mL tubes and centrifuged at 3,000 rpm for 10 min. The supernatant was filtered through a tea strainer, and approximately 35 mL of filtrate was collected for analysis.

Taste characteristics were measured using a taste sensing system (TS-5000Z, Intelligent Sensor Technology, Inc., Atsugi, Japan; S2D Fig) at Saga University, following the general measurement principles and procedures described previously [34]. The reference sample for the taste sensing system measurements was a composite prepared by mixing surplus young edible culms collected from all sites, stored under frozen conditions (−20 °C) until immediately before analysis. Soluble solids content was measured on juice extracted from the samples using a portable digital refractometer (HI-96801, HANNA Instruments, Woonsocket, RI, USA), and recorded as Brix values.

### Environmental factors

Elevation (m a.s.l.) and slope angle (°) for each survey plot were calculated using geographic information system (GIS) data. Soil moisture content (%) was obtained from field surveys conducted in the Teshio Experimental Forest in July 2016 using a portable soil moisture sensor (TRIME-COMO^®^, Tohoku Electronic Industrial, Sendai, Japan). The soil moisture data were used to characterize relative differences in moisture conditions among study plots, which are partly influenced by topography-related water movement and drainage conditions, rather than to represent soil moisture conditions at the time of sampling in 2024. Elevation, slope angle, and soil moisture did not differ significantly between the two treatment groups (S2 Table). Detailed descriptions of the measurement methods used to estimate these environmental variables are provided in [31].

### Statistical analyses

Of the taste indices obtained from the taste sensing system, bitter aftertaste, astringency, umami, and umami richness, which were considered interpretable as human taste perceptions (see Results), were selected for analysis. In addition, Brix values were included as an indicator of sweetness. In some plots, the number of harvested bamboo shoots was insufficient for taste sensing measurements; consequently, complete data were available for 41 plots (21 control plots and 20 harvested plots), and plots with insufficient sample material were excluded from the analyses. Model assumptions were assessed by visual inspection of residual plots.

To explore the effects of harvesting treatment (harvested vs. control) and environmental variables (elevation, slope angle, and soil moisture) on each taste index, separate general linear models (GLMs) assuming a Gaussian error distribution with an identity link function were constructed with each taste index as the response variable, treating each study plot as an independent sampling unit.

Principal component analysis (PCA) was then conducted to evaluate multiple taste indices simultaneously. For PCA, all taste indices were standardized (mean = 0, variance = 1). Principal components with eigenvalues greater than 1 were retained and interpreted based on factor loadings; principal component scores were calculated for each study plot. Relationships between principal component scores and environmental variables were then analyzed using GLMs, with elevation, slope angle, soil moisture, and harvesting treatment as explanatory variables. Prior to model fitting, multicollinearity among environmental predictors was assessed using Pearson’s correlation coefficients and variance inflation factors (VIFs). Correlations among predictors were weak (|r| ≤ 0.249), and all VIF values were low (1.07–1.12), indicating no substantial multicollinearity. All statistical analyses were performed using JMP 18 (SAS Institute Inc., Cary, NC, USA), with a significance level of 5%.

## Results

The taste sensing system evaluated sourness, bitter aftertaste, astringency, umami, saltiness, bitterness, and umami richness. Of these, sourness, saltiness, bitterness, and astringency were not detected, and subsequent analyses therefore focused on umami, bitter aftertaste, astringency, and umami richness. Differences in stimulus intensity of less than 20% are considered imperceptible under Weber’s law [36]. In this system, a difference corresponding to 20% of the concentration of taste substances is defined as one unit, and differences of one unit or more are regarded as perceptible to humans [34].

Radar charts constructed separately for harvested and control plots revealed no clear visual differences between treatments (Fig 1), and no statistically significant differences were detected between treatments for any taste index (Table 1). In contrast, large variations in taste indices were observed within both harvested and control plots. Similarly, Brix values showed no significant differences between harvested and control plots, while substantial within-treatment variation was observed (Table 1).

**Table 1:** Results of general linear models (GLMs) examining the relationships between taste indexes and harvesting treatment/environmental variables. Adjusted *R*² and *P* values are shown for each model. Bitter aftertaste, astringency, umami, and umami richness were derived from taste sensing scores obtained using a taste sensing system, whereas sweetness was measured as Brix values using a refractometer. Statistically significant results are shown in bold.

Treatment/Environmental variables		Taste properties				
		Bitter aftertaste	Astringency	Umami	Umami richness	Sweetness (Brix value)
Harvest treatment	*R* ^2^	−0.008	−0.024	−0.023	0.023	−0.023
	*P*	0.423	0.961	0.166	0.163	0.701
Elevation	*R* ^2^	**0.309**	0.012	**0.184**	0.022	**0.404**
	*P*	**<0.001**	0.225	**0.002**	0.164	**<0.001**
Slope angle	*R* ^2^	**0.149**	−0.014	0.065	0.016	**0.154**
	*P*	**0.006**	0.530	0.053	0.197	**0.007**
Soil moisture	*R* ^2^	0.048	−0.016	−0.001	−0.020	0.021
	*P*	0.087	0.557	0.332	0.665	0.186

**Fig 1:**
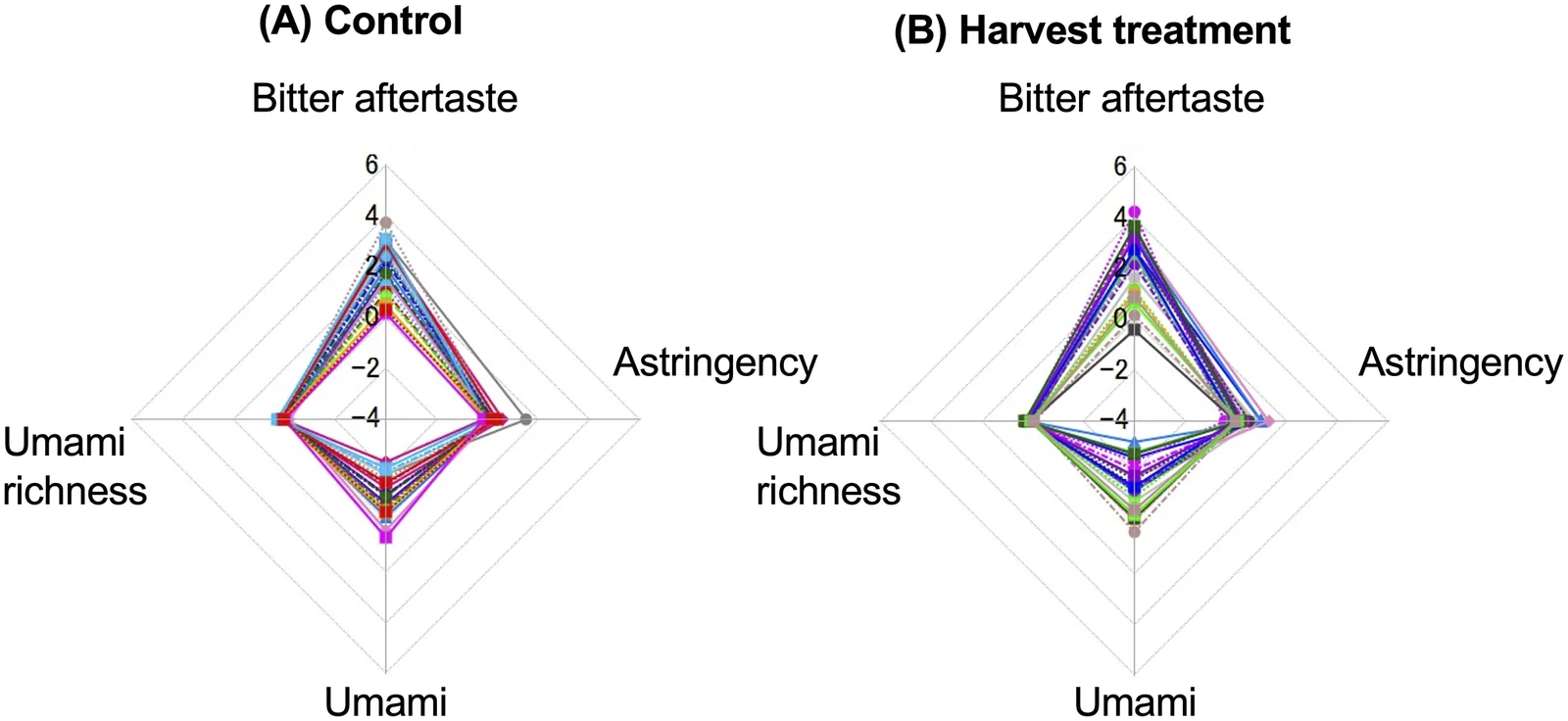
Radar charts of taste indices obtained using a taste sensing system. Each radar chart displays all plots within each treatment group (A: control; B: harvested), with each polygon representing a single plot, and four taste indices (bitter aftertaste, astringency, umami, and umami richness). Taste indices are expressed relative to a composite reference sample.

Soil moisture had no significant effect on any taste index (Table 1). In contrast, elevation showed significant associations with bitter aftertaste, umami, and sweetness, while slope angle showed significant associations with bitter aftertaste and sweetness (Table 1). Specifically, plots at higher elevations on steeper slopes tended to exhibit lower bitter aftertaste and higher sweetness and umami (Fig 2).

**Fig 2:**
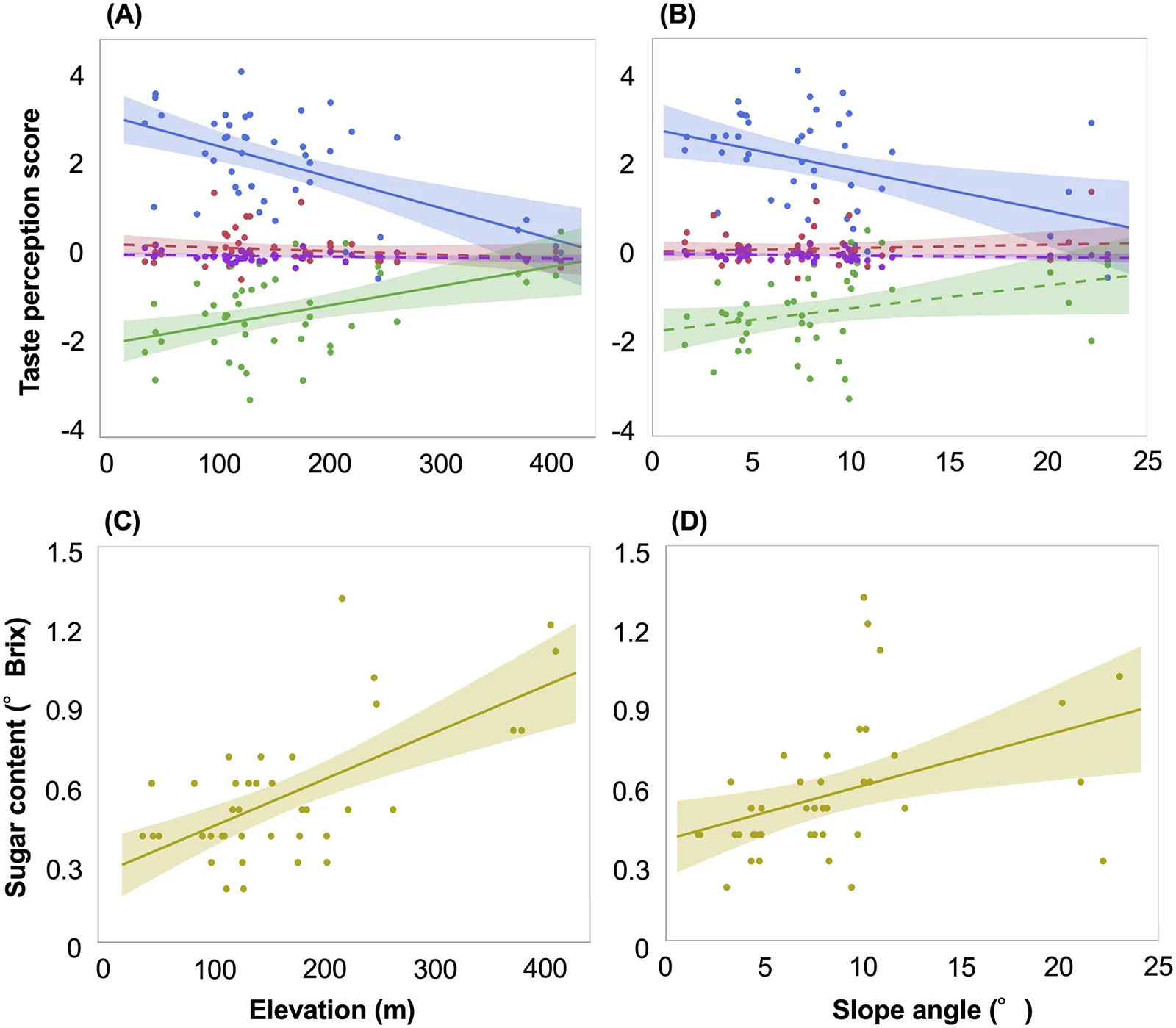
Effects of elevation and slope angle on bamboo shoot taste scores and sugar content. Points represent individual sampling plots. Solid lines represent statistically significant relationships (p < 0.05) and dashed lines indicate non-significant relationships (p > 0.05) based on separate general linear models (GLMs). Panel colors in (A) and (B) denote taste indices: blue, bitter aftertaste; red, astringency; green, umami; and purple, umami richness.

PCA of multiple taste indices yielded two principal components (Fig 3). The first principal component (PC1) was primarily driven by bitter aftertaste, umami, and sweetness; higher PC1 scores were associated with higher bitter aftertaste and lower umami and sweetness (factor loadings: bitter aftertaste = +0.918, umami = −0.968, sweetness = −0.916). PC2 scores were mainly driven by astringency and umami richness, with higher PC2 scores indicating higher astringency and lower umami richness (factor loadings: astringency = +0.845, umami richness = −0.521) (Fig 3).

**Fig 3:**
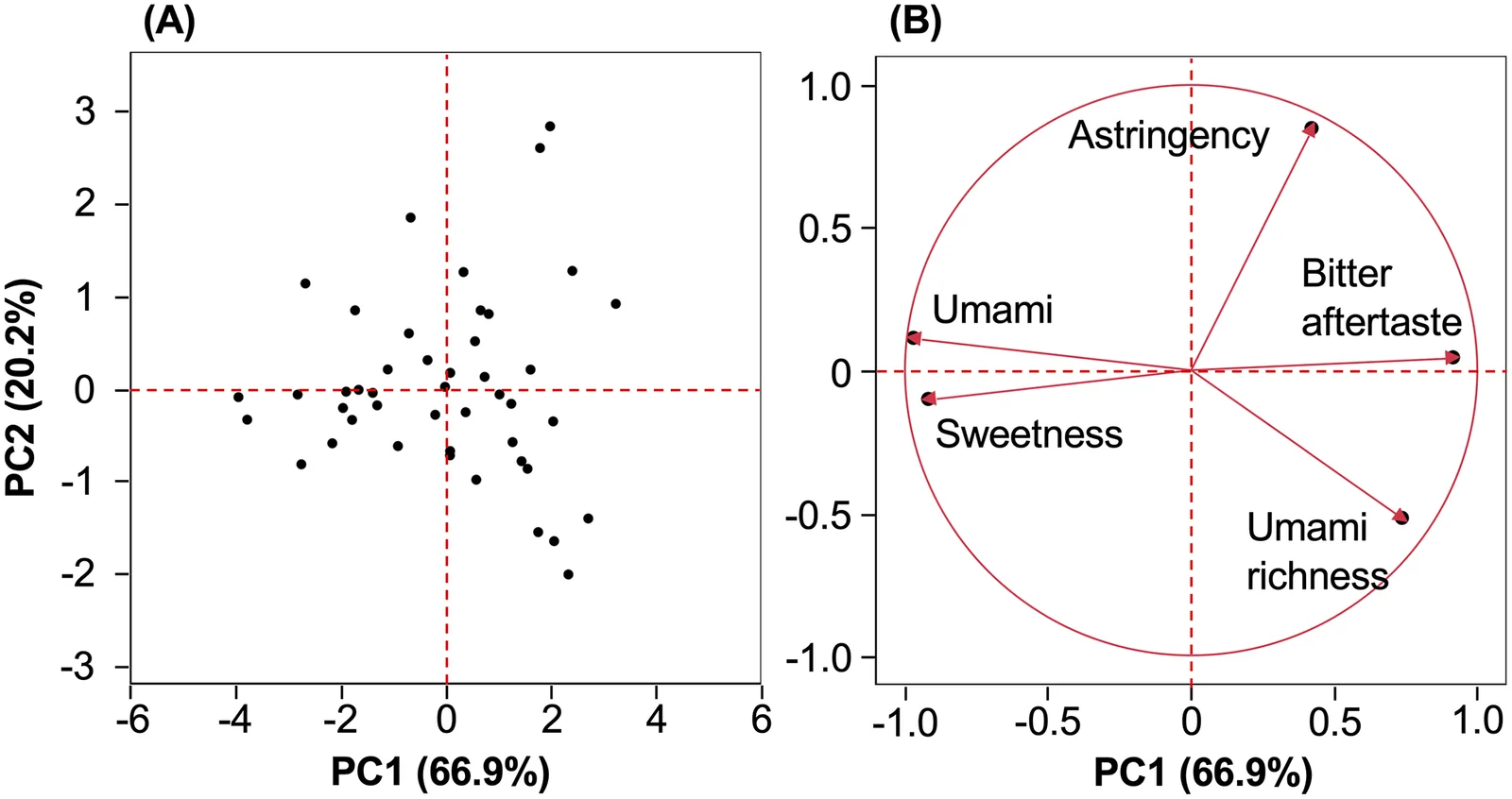
Principal component analysis (PCA) of multiple taste indices. (A) Score plot of the first (PC1) and second (PC2) principal components. (B) Factor loadings of each taste index in the principal component space. The analysis included umami, bitter aftertaste, astringency, umami richness, and sweetness (sugar content; °Brix). The proportion of variance explained by each principal component is indicated on the axis labels.

GLMs examining the relationships between principal component scores and environmental variables showed that PC1 scores were significantly associated with elevation (Table 2), decreasing at higher elevations (Fig 4). No significant relationships were detected between PC2 scores and any environmental variable (Table 2).

**Table 2:** Relationships between principal component (PC) scores and environmental variables examined using general linear models (GLMs).

PC	Predictor	Estimate	SE	*t* value	*p* value
PC1	Intercept	2.665	1.427	1.87	0.071
	Harvest treatment	0.233	0.254	0.92	0.365
	Elevation	−0.008	0.003	−2.89	**0.007**
	Slope angle	−0.082	0.049	−1.68	0.103
	Soil moisture	−0.024	0.044	−0.55	0.585
PC2	Intercept	0.199	1.000	0.20	0.843
	Harvest treatment	−0.002	0.178	−0.01	0.989
	Elevation	−0.001	0.002	−0.69	0.493
	Slope angle	0.040	0.034	1.18	0.247
	Soil moisture	−0.008	0.031	−0.26	0.793

Estimates are regression coefficients from GLMs. SE indicates standard error. Significant effects (p < 0.05) are shown in bold.

**Fig 4:**
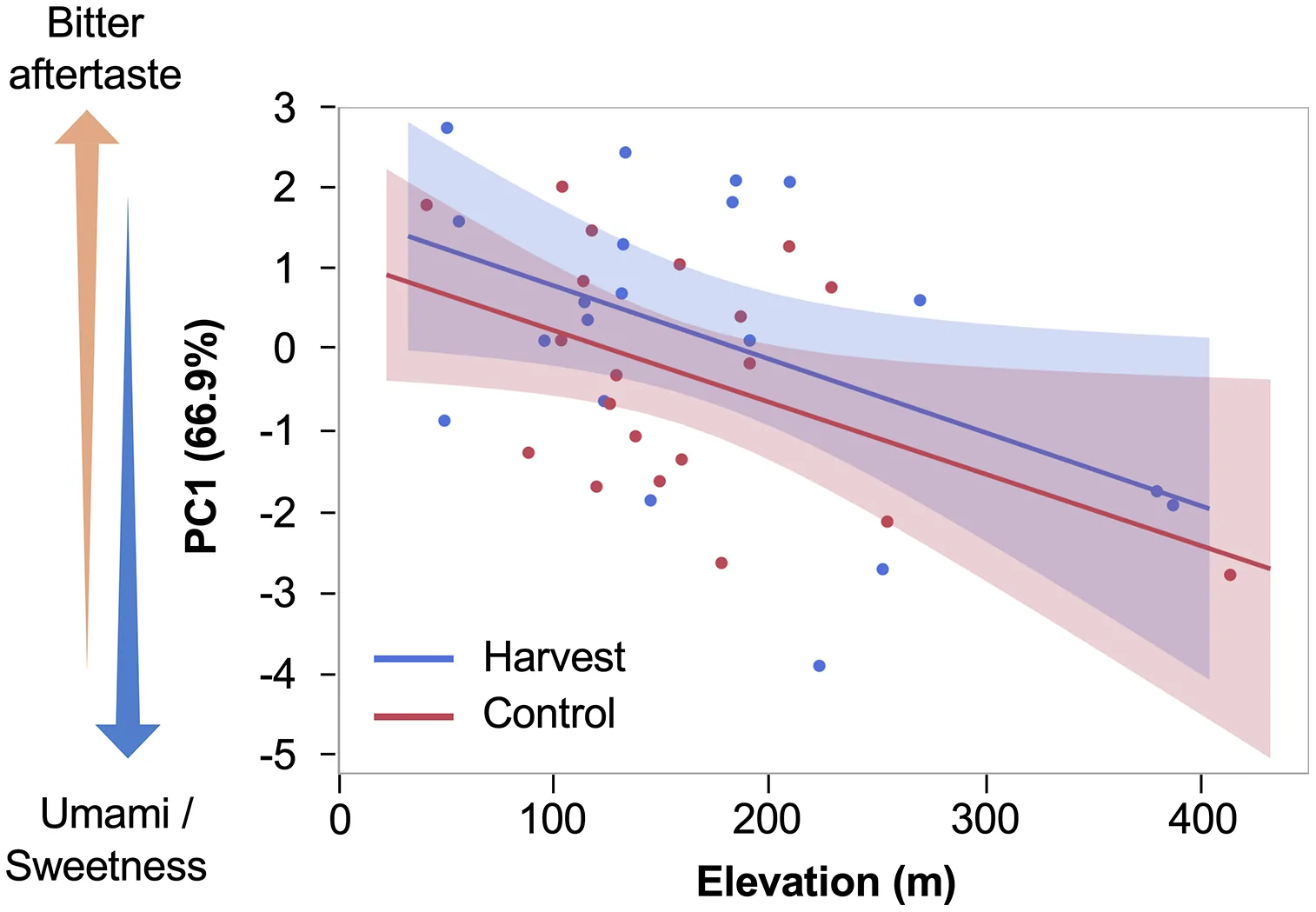
Relationship between elevation and the first principal component (PC1) derived from PCA. Higher PC1 values indicate stronger bitter aftertaste, whereas lower PC1 values indicate higher umami and sweetness (sugar content; °Brix). Points represent individual sampling plots; red indicates control plots and blue indicates harvested plots. Regression lines are shown separately for each treatment.

## Discussion

This study demonstrates that the taste characteristics of edible young culms of *S. kurilensis* are shaped primarily by environmental gradients rather than by human-harvesting. These findings suggest that spatial variation in taste reflects ecological responses to topographic conditions, highlighting the importance of environmental filtering in determining the quality of wild edible plant resources.

In many plant species, herbivory and mechanical damage are known to induce responses that alter the chemical composition of plant tissues, including secondary metabolites [17–19]. Because the chemical composition of plant tissues contributes to their taste characteristics [37,38], harvesting, which involves the removal of plant tissues, may also act as a form of damage and potentially affect the taste characteristics of subsequently produced edible tissues. Indeed, previous studies [20,27] reported that harvesting young culms of *S. kurilensis* resulted in a compensatory increase in the number of young culms emerging in the following year, indicating that this species responds to harvesting by altering subsequent young culm production. Katayama [20] reported no significant effect of harvesting on taste based on sensory evaluation. Similarly, in the present study using a taste sensing system, no clear effect of harvesting on the taste characteristics of young culms was detected after repeated annual harvesting since 2019. These findings suggest that, although the response of *S. kurilensis* to harvesting is evident in young culm production, its effects may not necessarily extend to taste characteristics. One possible explanation is that the effects of harvesting, although sufficient to induce a compensatory response in young culm production in the following year, may not have been strong enough to alter the chemical compounds underlying taste characteristics to a detectable extent. It is also possible that, even if harvesting-induced damage elicited a transient chemical response, this response did not persist sufficiently to affect subsequently produced young culms.

Previous studies on *Sasa* spp. focused primarily on quantitative aspects such as the production and size of edible young culms [27,31], with little attention to how qualitative traits such as taste characteristics are affected by environmental factors. Our findings extend these previous studies by showing that environmental gradients associated with elevation and slope influence not only the quantity and morphology of edible young culms but also their taste characteristics. Here, we interpret these findings within two frameworks: (1) a cold-acclimation framework, in which low-temperature conditions at higher elevations influence taste characteristics through the accumulation of sugars and free amino acids; and (2) a morphology-mediated resource allocation framework, in which slope steepness affects resource allocation via changes in the morphology of parent culms and edible young culms, thereby altering taste characteristics.

### Cold-acclimation framework

The elevated sweetness and umami observed at higher elevations (Fig 2A and 2C) may be explained by physiological acclimation to low-temperature environments. Low-temperature stress in plants is broadly categorized into freezing stress, which involves ice formation, and chilling stress, which occurs under non-freezing conditions generally between 0 and 10°C [39]. Freezing stress arises from damage to cellular structures associated with ice formation. Under natural conditions, extracellular freezing predominates, and the resulting water potential gradient between extracellular ice and the cell interior draws water out of cells, so that freezing effectively acts as a form of dehydration stress [40,41]. Even under chilling conditions without freezing, impairments in membrane function and metabolic disturbances have been reported [39].

As an adaptive response to both freezing and chilling stress, cold-acclimated plants accumulate low-molecular-weight soluble compounds such as soluble sugars and amino acids intracellularly [41]. Soluble sugars including sucrose and glucose contribute to freezing tolerance through osmotic adjustment, depression of the freezing point, and stabilization of cellular membranes and proteins [39,41]. Increases in soluble sugars under low-temperature conditions have been reported in cabbage and potato tubers [42,43]. Low-temperature stress also promotes the accumulation of free amino acids such as glutamate and asparagine [44]. Because glutamate is a principal component of umami, the accumulation of free amino acids under cold acclimation may have contributed to the increase in umami observed in this study.

A negative correlation was observed between bitter aftertaste and both umami and sweetness (Brix) (Fig 3). Interpreting this relationship requires consideration of both the response characteristics of the taste sensing system and metabolic balances within plant tissues. The taste score generated by the taste sensing system is not determined by a single chemical compound, but is quantified based on patterns of potential changes resulting from the collective interactions between lipid polymer membranes and multiple components [32,33], including phenolics, alkaloids, and glycosides, which contribute to bitterness [37,38]. Bitter aftertaste should therefore be interpreted as an integrated indicator of the overall chemical composition and relative balance of components in the sample.

At least two possible explanations can be proposed for this negative correlation. First, the accumulation of soluble sugars and free amino acids during cold acclimation may have enhanced the sensor response to sweetness and umami, thereby relatively reducing the contribution of bitter aftertaste, even if the absolute concentration of bitter compounds remained unchanged. Second, as a trade-off in resource allocation within the plant, the prioritization of growth, storage, and cold tolerance may have promoted the accumulation of primary metabolites (sugars and free amino acids), while suppressing the synthesis of compounds responsible for bitter aftertaste. Many such compounds, including phenolics, alkaloids, and glycosides, are secondary metabolites involved in herbivore defense and stress responses [45], the biosynthesis of which often draws on primary metabolites such as sugars and amino acids as carbon and nitrogen sources [46,47].

Although chemical components such as sugars and amino acids in edible young culms were not directly measured in this study, the observed increases in sweetness and umami are consistent with the general framework of cold acclimation, given the well-established physiological response whereby soluble sugars and amino acids accumulate as an adaptation to low-temperature environments at higher elevations.

### Morphology-mediated resource allocation framework

Separate GLM analyses showed that study plots with steeper slopes tended to exhibit higher sweetness and umami and lower bitter aftertaste in young culms (Fig 2B and 2D). However, the GLM analysis of PCA scores indicated that slope angle did not significantly affect PC1 (a taste axis characterized by high bitter aftertaste and low umami and sweetness); only elevation was identified as a significant explanatory variable (Table 2). Therefore, the relationship between slope angle and taste characteristics is not consistently supported when taste is evaluated in an integrated manner. Nevertheless, because consistent trends were observed in the separate GLM analyses, the association of slope angle with the taste characteristics of edible young culms cannot be ruled out.

In the same study plots, Katayama [31] showed that on steeper slopes, the density of older culms decreases while culm diameter increases, and that these morphological traits determine both the number and diameter of edible young culms produced in the following year. It was further suggested that the formation of thicker culms in steep environments is driven by mechanical constraints, as greater structural support is required to withstand loads from self-weight, wind, and snow. Thicker culms are structurally advantageous under these conditions, and this morphological characteristic is reflected in the production of relatively thicker edible young culms. How culm thickness relates to the amount of resources accumulated within edible young culms remains unclear, however, the strong influence of slope angle on *Sasa* culm morphology [31], together with the tendency for higher sweetness and umami at steeper sites, suggests a possible linkage between morphological traits and taste characteristics in edible young culms.

In *Sasa* spp., the clonal system, including belowground structures, stores resources and distributes them through rhizome networks [48–50]. Edible young culms photosynthesize little during early development and grow primarily on non-structural carbohydrates (NSC) supplied from mature culms and belowground organs, which function as strong sinks within a source–sink system [49,51]. NSC comprises starch and soluble sugars, and NSC stored in rhizomes and mature culms is translocated primarily as soluble sugars from source organs to sink tissues (i.e., edible young culms) [49]. These sugars serve as the primary energy source for early growth and underlie sweetness (Brix). *Sasa* stands that produce thicker edible young culms may also be in a state of higher resource storage and translocation capacity at the clonal level, including belowground organs. For example, *Sasa* stands that have recovered from forestry disturbance and attained larger stand sizes tend to produce relatively thicker edible young culms [28]. Future studies should quantitatively compare the concentrations of sugars and free amino acids between thicker and thinner edible young culms and analyze morphological traits, chemical composition, and taste characteristics in an integrated framework to empirically test this hypothesis.

### Spatially explicit interpretation of productivity and taste based on topography

Katayama [31] showed that the density and diameter of edible young culms produced in a given year are determined by the density and diameter of parent culms, respectively. In addition, a trade-off was observed between the density and thickness of edible young culms, indicating that two contrasting production structures can occur: one characterized by the production of numerous thin edible young culms, and another by fewer but thicker edible young culms. Topographic analysis further revealed that, in flat areas, edible young culms are produced in large numbers but are relatively thin, resulting in low production of the thick, high-food-value (valuable culms). In steep slope environments, thicker edible young culms are more likely to be produced, but their numbers are limited, resulting in lower overall production.

Integrating these findings with the present results, the following process is suggested: (1) thicker culms are more likely to form on steep slopes; (2) this morphological characteristic is reflected in the production of thicker edible young culms, potentially leading to greater accumulation of stored resources; and (3) consequently, taste characteristics are enhanced. Elevation was identified as a significant explanatory variable in both the separate GLMs for individual taste indices and the GLM of PCA scores, suggesting that temperature differences associated with elevation may influence taste characteristics through the accumulation of sugars and free amino acids. Thus, slope angle may influence taste characteristics primarily through morphological development and production structure, whereas elevation may act through physiological and metabolic processes mediated by temperature. These results suggest distinct pathways by which slope and elevation influence taste characteristics.

Together, these findings enable spatially explicit predictions of where edible young culms with particular taste characteristics are produced and in what quantities, based on topographic conditions. In areas characterized by steep slopes and high elevation, few edible young culms are produced, but they are likely to be thicker and richer in sweetness and umami. Conversely, in areas with gentle slopes or at low elevation, larger numbers are produced, but their taste characteristics tend to be relatively lower. Such spatial predictions provide a useful perspective for understanding foraging practices of local communities and the resource value of *Sasa* forests, as well as for evaluating how the spatial structure of high-quality edible young culm production may change under climate change. For example, if warming reduces low-temperature conditions at higher elevations, the accumulation of sugars and free amino acids may decline, potentially reducing the environments favorable for producing edible young culms rich in sweetness and umami.

### Conclusions and future directions

This study quantitatively evaluated how harvesting and topographic conditions influence the taste characteristics of edible young culms of *S. kurilensis*. By integrating objective taste measurements with environmental analyses, this study demonstrated that spatial variation in taste characteristics is more closely associated with topographic conditions than with harvesting treatment. The novelty of this study lies in integrating taste characteristics into the production structure of edible young culms, which was previously discussed mainly in terms of production and morphological traits, and in quantitatively linking their spatial variation to topographic conditions. These findings address an important knowledge gap regarding the environmental determinants of taste characteristics in wild edible plants. More broadly, this expands the evaluation of wild edible plant resources from a purely quantitative perspective to one that also incorporates resource quality and its spatial variation. Such an approach provides a basis for sustainable resource management and for predicting how climate change may alter the distribution of high-quality edible resources.

However, several challenges remain for a more rigorous understanding of the relationships identified in this study. First, the validity of the spatial predictions identified in this study needs to be tested. Because the study is based on results from a single region, it is necessary to develop spatial prediction models based on topographic information and to evaluate how well predicted taste characteristics correspond to observed values. The soil moisture data used here were derived from past measurements and may not directly reflect conditions at the time of sampling; however, because soil moisture had no significant effects on taste characteristics, this temporal mismatch is unlikely to substantially affect the main conclusions. Second, interannual variation needs to be assessed; long-term observations across multiple years are needed to determine whether the relationships between topographic conditions and taste characteristics are consistent across years or specific to particular climatic conditions. Third, the physiological and morphological mechanisms proposed in this study require empirical validation. In particular, it is necessary to quantify the relationship between elevation and actual temperature conditions and to determine, through direct measurements of chemical components, whether low-temperature exposure promotes the accumulation of sugars and free amino acids. It is also important to examine whether temperature conditions during the growing season or the overwintering period more strongly shape taste characteristics. Addressing these challenges will advance a more causal and predictive understanding of the relationships between topographic conditions and taste characteristics, and provide a framework for predicting how climate warming may alter the spatial structure of environments that have traditionally favored the production of edible young culms with high palatability.

## Supporting information

S1 Fig Map of study plots in the Teshio Experimental Forest, Hokkaido University. Solid red circles indicate plot locations; some plot symbols overlap due to plot proximity. The plots were originally established in 2013 (n = 50), but six plots had been lost by 2023 and three were excluded from the analysis due to an insufficient number of harvested edible young culms.
(DOCX)

S2 Fig Overview of the study species and analytical instruments. (A) *Sasa kurilensis* at the study site. (B) Harvested edible young culms of *S. kurilensis*. (C) Pretreated edible young culm samples sealed in glass jars. (D) Taste sensing system (TS-5000Z, Intelligent Sensor Technology, Inc.) at Saga University.
(DOCX)

S1 Dataset Raw data used for statistical analyses in this study. (XLSX)

S1 Table Young culm abundance in plots subsequently assigned to control and harvesting treatments before the harvesting experiment. (XLSX)

S2 Table Comparison of environmental conditions between control and harvesting plots. (XLSX)
