# Supplementary material for: Environmental drivers of taste characteristics in wild edible plants: Effects of topographic gradients and human harvesting evaluated using dwarf bamboo culms
Source: PLoS One. 2026 Oct 28;21(10):e0360962. doi: 10.1371/journal.pone.0360962 (PMC13716176; doi:10.1371/journal.pone.0360962)
Supplement: Supplementary file 1 [file pone.0360962.s001.docx]

**S1 Fig. Map of study plots in the Teshio Experimental Forest, Hokkaido University.** Blue circles indicate control plots, and red circles indicate harvest plots, where harvesting treatments have been conducted since 2019. The grayscale background represents elevation, with darker areas indicating higher elevations.
