# Supplementary material for: Environmental drivers of taste characteristics in wild edible plants: Effects of topographic gradients and human harvesting evaluated using dwarf bamboo culms
Source: PLoS One. 2026 Oct 28;21(10):e0360962. doi: 10.1371/journal.pone.0360962 (PMC13716176; doi:10.1371/journal.pone.0360962)
Supplement: Supplementary file 2 [file pone.0360962.s002.docx]

**S2 Fig. Overview of the study species and analytical instruments.** A) *Sasa kurilensis* at the study site. B) Harvested edible young culms of *S. kurilensis*. C) Pretreated edible young culm samples sealed in glass jars. D) Taste sensing system (TS-5000Z, Intelligent Sensor Technology, Inc.) at Saga University.
